# P-960. Evaluation of a discharge-focused stewardship intervention in a stepped wedge cluster randomized trial across 10 hospitals

**DOI:** 10.1093/ofid/ofaf695.1162

**Published:** 2026-01-11

**Authors:** Daniel Livorsi, Alyssa Thompson, Angela Hoelscher, Kailye Chu, Elizabeth Neuner, Yvonne Burnett, Teri Hopkins, Elizabeth Walter, Rohini Dave, Ravi Tripathi, Haylie Lohmar, Andrew Dysangco, Kelly M Percival, Dilek Ince, Jessica Kolkmeyer, Helen Newland, Joshua Hendrix, Gosia Clore, Cody Poe, Amy O’Shea, DeShauna Jones, Emily E Chasco, Joseph Tholany, Kunatum Prasidthrathsint, Erin Rachmiel, Jahnavi Bongu, Alice Bewley, Kevin Hsueh

**Affiliations:** University of Iowa Carver College of Medicine, Iowa City, IA; Barnes Jewish Healthcare, St. Louis, Missouri; Barnes Jewish Healthcare, St. Louis, Missouri; Barnes Jewish Healthcare, St. Louis, Missouri; Barnes-Jewish Hospital, St. Louis, Missouri; SSM Health Saint Louis University Hospital, St. Louis, MO; 1. South Texas Veterans Healthcare System, San Antonio TX 2. UT Long School of Medicine, San Antonio TX 3. UT Austin College of Pharmacy, Austin TX, San Antonio, Texas; Audie L. Murphy Memorial Veterans Hospital, San Antonio, Texas; VA Maryland Health Care System, Baltimore, MD; University of Maryland School of Medicine, Baltimore, Maryland; Richard Roudebush VAMC, Indianapolis, Indiana; Atlanta VA Medical Center, Atlanta, Georgia; University of Iowa Health Care, Iowa City, IA; University of Iowa Hospitals & Clinics, Iowa City, Iowa; BJC Christian Hospital, St. Louis, Missouri; BJC HealthCare, St. Louis, Missouri; Barnes Jewish Healthcare, St. Louis, Missouri; University of Iowa, Iowa City, Iowa; University of Iowa Carver College of Medicine, Iowa City, IA; University of Iowa Carver College of Medicine, Iowa City, IA; University of Iowa, Iowa City, Iowa; University of Iowa, Iowa City, Iowa; University of Iowa Carver College of Medicine, Iowa City, IA; University of Iowa Carver College of Medicine, Iowa City, IA; Washington University (St. Louis), St. Louis, Missouri; Washington University (St. Louis), St. Louis, Missouri; Washington University School of Medicine in St. Louis, St. Louis, Missouri; Washington University in St. Louis, Saint Louis, MO

## Abstract

**Background:**

We evaluated whether an antibiotic stewardship bundle, which included audit-and-feedback, could reduce antibiotic overuse at hospital discharge.

**Figure:**
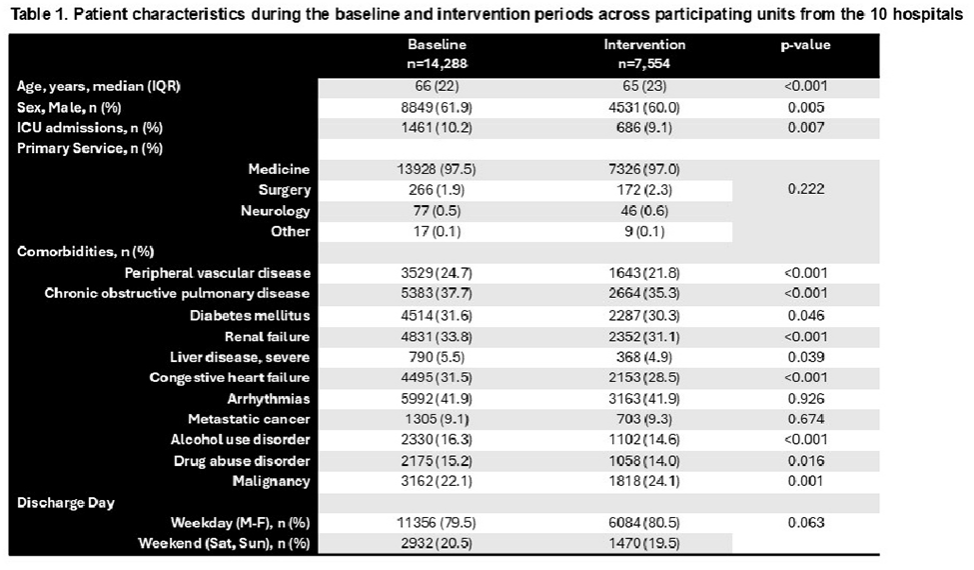

**Figure:**
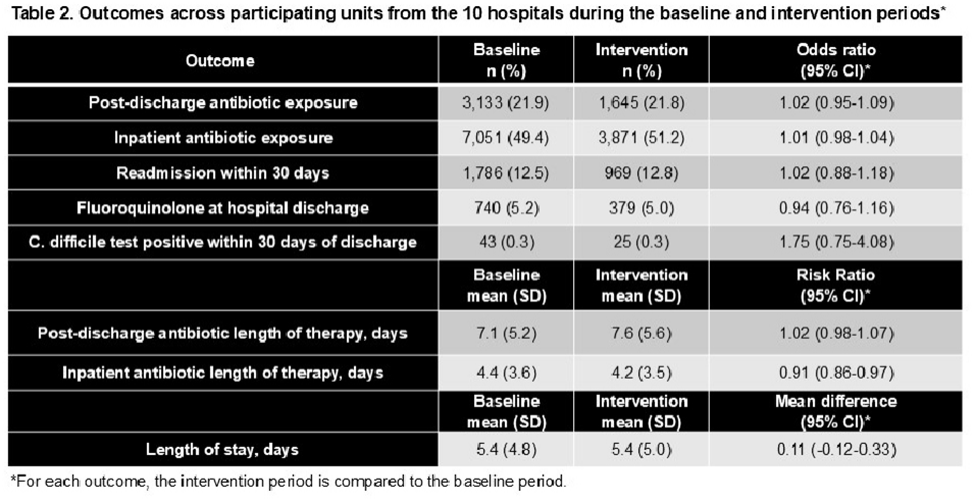

**Methods:**

We performed a stepped-wedge cluster randomized trial across participating units at 10 hospitals to evaluate the effect of a discharge-focused stewardship bundle. The trial ran from 12/5/22-11/17/23. After a 24-week baseline period, one hospital crossed into the intervention arm every 2 weeks. The intervention consisted of a) disseminating institutional guidelines for oral antibiotic step-down therapy; and b) prospective audit-and-feedback on inpatients receiving antibiotics who had an anticipated discharge in the next 48 hours. The primary outcome was post-discharge antibiotic use. Secondary outcomes included inpatient antibiotic use, length-of-stay, and readmissions. After the intervention ended, providers and stewardship personnel from each hospital were surveyed and interviewed, respectively, to assess acceptability and feasibility.

**Figure:**
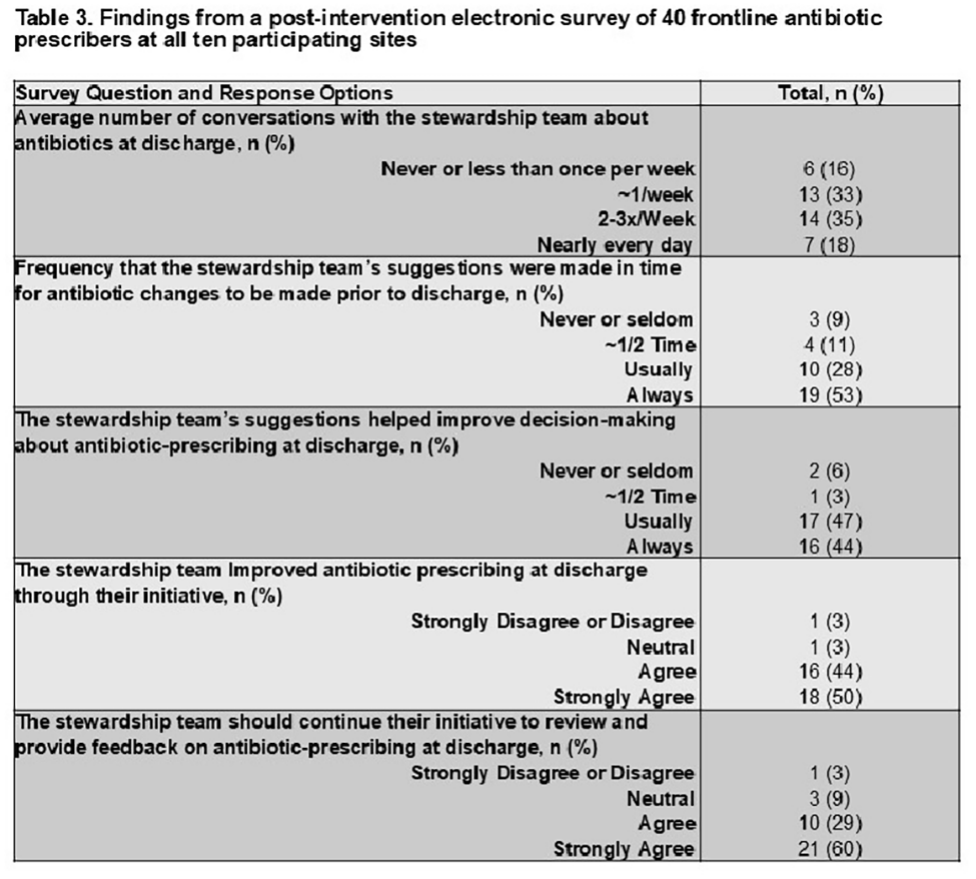

**Figure:**
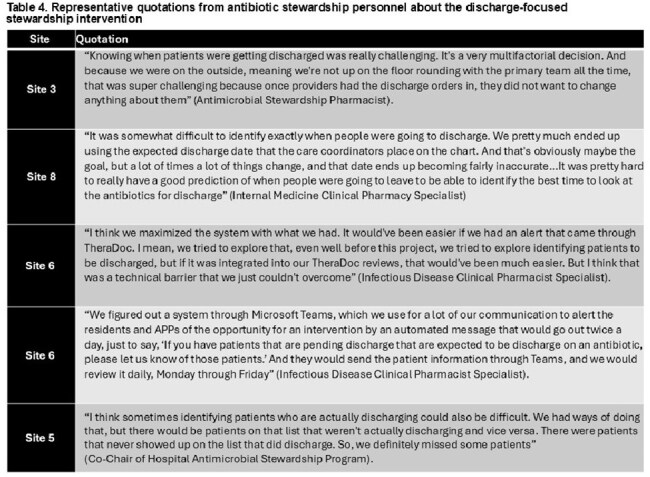

**Results:**

There were 21,782 patient-admissions included (14,228 baseline period; 7,554 intervention period). Median age was 66 years, with 61% male (Table 1). At the hospital-level, the average number of patients audited per week was 20; one-quarter of audits led to feedback. There were 3,133 (21.9%) patients prescribed post-discharge antibiotics at baseline compared to 1,645 (21.8%) during the intervention (OR 1.02; 95% CI 0.95-1.09). The mean post-discharge duration was 7.1 days (SD 5.2) at baseline compared to 7.6 days (SD 5.6) during the intervention (RR 1.02; 95% CI 0.98-1.07). Inpatient antibiotic duration was shorter during the intervention (RR 0.91; 95% CI 0.86-0.97), but there was no difference in length-of-stay and readmissions (Table 2). One hundred twelve inpatient providers were sent a post-intervention survey; 40 (35.7%) responded; 94% felt that the initiative improved antibiotic-prescribing at discharge (Table 3). However, many stewardship teams reported difficulty in accurately identifying when patients would be discharged (Table 4).

**Conclusion:**

An antibiotic stewardship bundle, which included audit-and-feedback, did not reduce antibiotic use at discharge. The bundle was acceptable to providers but difficult for stewardship teams to implement.

**Disclosures:**

Yvonne Burnett, PharmD, BCIDP, InflaRx: Honoraria|Melinta Therapeutics: Honoraria

